# Mito-oncology agent: fermented extract suppresses the Warburg effect, restores oxidative mitochondrial activity, and inhibits in vivo tumor growth

**DOI:** 10.1038/s41598-020-71118-3

**Published:** 2020-08-25

**Authors:** Gyula Bencze, Szilvia Bencze, Keith D. Rivera, James D. Watson, Mate Hidvegi, Laszlo Orfi, Nicholas K. Tonks, Darryl J. Pappin

**Affiliations:** 1 Cold Spring Harbor Laboratory, Cold Spring Harbor, NY 11724 USA; 2 American Biosciences, Inc, Blauvelt, NY 10913 USA; 3 Department of Pharmaceutical Chemistry, Semmelweis University, Budapest, 1085 Hungary; 4 Jewish Theological Seminary—University of Jewish Studies, Budapest, 1084 Hungary

**Keywords:** Cancer metabolism, Protein-protein interaction networks, Targeted therapies, Metabolomics, Energy metabolism

## Abstract

Mitochondrial dysfunction and significant changes in metabolic pathways accompany cancer development and are responsible for maintaining the tumor microenvironment. Normal mitochondria can trigger intrinsic apoptosis by releasing cytochrome c into the cytosol. The survival of malignant cells highly depends on the suppression of this function. We validated that A250, a highly purified fraction of fermented wheat germ extract (FWGE), increases the carbon flux into the mitochondria, the expression of key elements of the Krebs cycle and oxidative phosphorylation (OXPHOS). The increased respiratory chain activity is related to the mitochondria’s ability to release cytochrome c into the cytosol, which triggers the apoptotic cascade. The 68% tumor growth inhibitory effect observed in the murine melanoma study is related to this effect, as proteomic analysis validated similar changes in mitochondrial protein levels in the isolated tumor tissue samples. Blood count data indicated that this effect was not accompanied by general toxicity. This study is significant, as it shows that a highly concentrated form of FWGE is an effective agent that increases normal mitochondrial functionality. The lack of hepatotoxic and general toxic effects makes A250 an excellent candidate targeting mitochondria function in cancer therapy.

## Introduction

Aerobic glycolysis is a characteristic of cancer cells, as observed by Otto Warburg almost a century ago^1^. Metabolic reprogramming is considered a hallmark of cancer cells and is an essential requirement to provide sufficient nucleotides, proteins, and lipids that can double a cell’s biomass^2^. Unlike quiescent cells, proliferating cells are in much greater need of reduced carbon and nitrogen; therefore, rather than maximizing the production of ATP through oxidative phosphorylation (OXPHOS), they use mitochondrial enzymes in the synthesis of anabolic precursors. Furthermore, proliferating cells do not accumulate cytosolic nicotinamide adenine dinucleotide (NADH) and have reduced ATP production by converting excess pyruvate to lactic acid^3^. The conversion of pyruvate to lactic acid by lactate dehydrogenase A (LDH-A) is the key switch between OXPHOS and glycolysis, as LDH-A significantly reduces the flux of glucose-derived carbon into mitochondria^4^. This reprogrammed metabolism negatively affects essential mitochondrial functions, such as cell cycle regulation and mitochondrial-mediated apoptosis, contributing to tumorigenesis and cancer cell survival. Thus, it is not surprising mitochondrial and metabolic dysfunction is a hallmark of cancer^5^. Inhibition of the Warburg effect by increasing the flux of carbon back to mitochondria through the inhibition of either LDH-A or pyruvate dehydrogenase kinase (PDK) is a selective and efficient way of targeting cancer cell proliferation^6–8^.

Based on the publications of Nobel laureate Albert Szent-Gyorgyi in the early 1980s^9,10^, fermented wheat germ extract (FWGE) was first studied as a potential anticancer agent in the 1990s^11^. The effect of FWGE on glycolysis was described in 2001^12,13^, and its in vivo anti-cancer effects have been described many times^14^. Its supportive effects in cancer treatment have been validated in several small-scale clinical trials^15–22^.

In this study, we utilize a concentrated fraction of FWGE purified with bioassay-guided fractionation. We describe the effect of this fraction on mitochondrial function as a potential mode of action underlying its tumor growth inhibitory effect, as proteomic data showed increased levels of mitochondrial proteins in vitro and in vivo, metabolomics data demonstrated increased pyruvate processing in the mitochondria, and the observed beneficial changes in oxygen consumption and extracellular acidification indicate more active respiration and suppression of the Warburg effect. The lack of hepatotoxicity and general toxicity was confirmed with whole blood panel and pathological analyses.

## Materials and methods

### Isolation of active components

For active component isolation, 10 g of whole standardized, commercially available FWGE, provided by American Biosciences Inc. (Blauvelt, NY), was washed four times with 30 mL of methanol (Sigma). With each wash, the suspension was vortexed and sonicated for 5 min. The insoluble components were centrifuged at 4000 rpm, and the supernatant was collected after each step, merged, and stored at − 80 °C overnight. The precipitated components were micro-filtered, and the methanolic solution was evaporated to dryness under vacuum at 40 °C. Fraction A2 represented 35–45% of whole FWGE biomass.

Fraction A2 was dissolved in 80 mL of water and passed through an HLB solid-phase extraction (SPE) column (Waters). After two washes with water, the bound components were eluted with methanol, and the solvent was removed under vacuum. The sample was dried completely in a speed-vac. Fraction A250 was 2.7–3.2% of whole FWGE biomass.

To verify reproducibility, A250 was dissolved in dimethyl-sulfoxide (DMSO, Sigma Aldrich) at a 10 mg/mL concentration, and 20 μL was injected in a Phenomenex C18(2) (150 × 4.6 mm, 3 µm) column connected to a Waters HPLC system with a diode array detector. The concentration of acetonitrile (with 0.1% formic acid) was increased up to 30% over 15 min, followed by a 95% wash for 2 min.

### Cell culture

Unless otherwise indicated, cancer cells were grown in Dulbecco’s modified Eagle’s medium (DMEM, Invitrogen) supplemented with 10% (v/v) fetal bovine serum (FBS) and 100 U/mL penicillin and 100 mg/mL streptomycin at 37 °C in a humidified atmosphere of 5% CO_2_. Before reaching confluence, the cells were washed with phosphate-buffered saline (PBS) and harvested with trypsin (Invitrogen). The cells were discarded after 7–8 passages, and fresh cells were thawed and cultured.

### In vitro proliferation assay

The cytotoxicity of A250 was tested on embryonic kidney (HEK-293 T), murine melanoma (B16F10), metastatic ovarian (PA-1), metastatic breast (MCF-7), colon carcinoma (HCT-116), and two metastatic prostate (PC-3 and DU145) cell lines. All cell lines used were from the in-house stock and at low passage number. The cells were seeded in culture media in a Falcon 96-well plate and allowed to grow for 24 h. A250 was dissolved at a 1 mg/mL concentration and diluted in 1:2 serial dilutions. The cells were incubated for 72 h, and the effect of Fraction A250 on cell viability was determined with 3-(4,5-dimethylthiazol-2-yl)-2,5-diphenyltetrazolium bromide (MTT) reagent (Sigma-Aldrich)^23^. The absorbance was read at 650 nm, and the background was subtracted. The data were analyzed using GraphPad Prism 5.1 software.

### Quantitative proteomic analysis

B16F10 murine melanoma cells were plated in a T175 Petri dish (BD Falcon) and synchronized in serum-free medium overnight. After 24 h, the medium was replaced with serum-rich DMEM containing A250 at a 65 µg/mL concentration. The cells were washed with ice-cold PBS after 24 h of incubation and lifted with a cell scraper. The cell pellet was then collected by centrifugation at 1500 rpm.

For isobaric tag for relative and absolute quantitation (iTRAQ) labeling, pellets were lysed in 1–2 mL of 8 M urea, 50 mM triethyl-ammonium bicarbonate (TEAB) (pH 8.5) 0.05% w/v ProteaseMAX (Promega) containing phosphatase inhibitor and protease inhibitor cocktails (P2850, P5726, P8340, Sigma) and lysed again by passing three times through a 21G needle and another three passes through a 25G needle on ice. The lysate solutions were then centrifuged at 14,000 g. Supernatants were collected and protein concentrations were determined by bicinchoninic acid (BCA) assay. Aliquots of 100 ug total protein from each sample were reduced and alkylated by sequential incubations with 5 mM tris(2-carboxyethyl)phosphine (TCEP) and 10 mM methyl-methane-thio-sulfonate (MMTS)^24^. The reaction mixtures were then precipitated by the addition of methanol/chloroform (2:1 v/v), and the pellets were reconstituted in 50 μL of 6 M urea plus 50 mM TEAB with sonication. An additional 50 μL of 50 mM TEAB with trypsin (final ratio 1:50 w/w enzyme/protein) was added, and digestion was allowed to proceed overnight at 37 °C. An additional 2-h incubation with a fresh aliquot of trypsin (1:200 w/w) was performed the next morning. The solution volumes were reduced to a final volume of ~ 20 μL in a speedvac, and 20 μL of 1 M TEAB solution was added to each sample. Next, 8-plex iTRAQ reagents (Applied Biosystems) were added to 70 μL ethanol and incubated as described previously^24^. After labeling, each solution was acidified by the addition of 3 μL trifluoroacetic acid (TFA), the separate fractions were combined, and the total mixture was dried in a vacuum.

### Electrospray mass spectrometry (MS)

The combined pool of labeled peptides was dissolved in 40 μL 3% acetonitrile/0.1% formic acid and loaded onto a triple-phase capillary trap column (250 μm ID) packed with 2 cm 5 μm Aqua C18 material, 3 cm 5 μm Luna SCX material, and an additional 3 cm 5 μm Aqua C18 material. Peptides were sequentially eluted by 16 separate salt steps ranging from 25 to 500 mM ammonium acetate and separated on a 14-cm analytical column packed with 3 μm Aqua C18 material. Peptides were eluted with a gradient from 5 to 45% B (90% acetonitrile, 0.1% formic acid) for 60 min, 45–80% B for 15 min, and 80% B for 8 min at a flow rate of 300 nL/min. Survey full-scan spectra were acquired in an Orbitrap XL mass spectrometer (Thermo) with a resolution of 15,000 and a mass range of 400–1800 m/z. The ion selection threshold was 500 counts for MS/MS. The four most intense ions were selected for consecutive high-energy collisional dissociation (HCD) and collision-induced dissociation (CID) scans. Acquisition parameters for HCD were set at a resolution of 7500, isolation width of 2 Da, normalized collision energy of 45%, and activation time of 40 ms. Acquisition parameters for CID were as follows: normalized collision energy of 35%, activation Q at 0.25, and activation time of 30 ms. Dynamic exclusion settings were a repeat count of 1 in 30 s, exclusion for 120 s, and an exclusion list size of 500. Target auto gain control (AGC) values for the linear ion trap and orbitrap were essentially as described previously^25^. The MS data were processed using Mascot software. More than 4000 proteins were detected, and their quantities were determined relative to the non-treated cells. The confidence intervals were determined from the dataset using the EasyFit software. The co-expression of significantly up- or downregulated proteins was determined by STRING network analysis^26^, and the related biological processes were identified by gene ontology (GO) analysis^27,28^.

### Immunoblotting

HEK-293 T transformed embryonic kidney cells were seeded into a 6-well plate and allowed to grow until reaching 70–80% confluence. The medium was replaced with fresh medium containing A250, and after 4 h of incubation, the cells were washed with cold PBS, scraped, and pelleted. To separate the cytosolic fraction from the heavy membrane fraction, the cells were resuspended and permeabilized in RIPA buffer containing 300 µg/mL digitonin. The lysates were vortexed and incubated for 5 min at 4 °C and then spun down, and the pellets were lysed with 0.2% Triton-X-100 and 0.3% NP-40 as described previously^29^. The proteins were separated on a Criterion precast 10–20% SDS polyacrylamide gel (BioRad) in the proper running buffer and transferred to a nitrocellulose membrane with an iBlot dry-transfer system (Invitrogen). The blots were blocked in TBS-T containing 5% milk powder and incubated with the primary antibodies from the Apoptosis Antibody Sampler Kit (Cell Signaling) in 5% bovine serum albumin (BSA) at 4˚C for 12 h.

### Mitochondrial respiration assay

We used an XF Mitochondrial Stress test kit (Seahorse Bioscience Inc.) with some modifications to measure the oxygen consumption rate (OCR) and extracellular acidification rate (ECAR). B16F10 murine melanoma cells were seeded into the lower 96-well plate of the XFe96 (Seahorse Bioscience) extracellular flux assay kit. The optimal seeding density was determined prior to the assay to be around 20,000 cells/well. The cells were washed, and the medium was replaced with glucose- and glutamine-free medium. The pre-treatment was started 1 h prior to the assay. The plate was incubated at 37 °C in an oxygen-rich atmosphere, while the upper plate was calibrated according to the kit protocol. The baseline oxygen level and pH of the extracellular space were measured three times in 20 min prior to the injection of glucose and glutamine (both from Sigma-Aldrich). Final concentrations of the standards were 25 mM glucose and 5 mM glutamine, while the concentrations of the modulators of mitochondrial function were 3.0 µM oligomycin, 0.25 µM FCCP, and 10 µM antimycin-A and rotenone.

### ^13^C tracer study

B16F10 murine melanoma cells were seeded into a 60-mm dish in triplicate and incubated overnight to reach 50–60% confluence. The media was removed, and the cells were washed with PBS. Isotope-labeled glucose was dissolved to the desired concentration in pyruvate-, glucose-, and glutamine-free medium containing 10% FBS. A250 was dissolved at a 60 μg/mL concentration in complete medium containing [U6-^13^C_6_]-glucose (> 99% purity and 99% isotope enrichment for each carbon position; Cambridge Isotope Labs). The plates were incubated for 24 h in a 5% CO_2_ atmosphere at 37 °C. At the end of the culture period, the cells were washed with ice-cold normal saline and overlaid with 50% methanol. The cells were then detached by scraping, and the suspension was transferred to an Eppendorf tube. The samples were then snap-frozen in liquid nitrogen. After three thaw–freeze cycles, the samples were spun down at 10,000 rpm, the supernatants were transferred into new tubes, and the solvent was evaporated with a sample concentrator. The completely dried samples were derivatized with Methoxamine (Thermo) prior to silylation with Tri-Sil HTP reagent (Thermo).

### Gas chromatography (GC)

The derivatized samples of the isotope tracer assay were resuspended in hexane and injected into an Agilent HP6890 connected to an HP 5937 GC–MS system. The temperature settings were as follows: GC inlet 230 °C, transfer line 280 °C, MS source 230 °C, MS Quad 150 °C. The metabolites were separated on a HP-5 capillary column with the following parameters: 30 m length, 250 μm diameter, and 0.25 μm film thickness. The MS data were collected with Agilent Mass Hunter software and analyzed using quantitative analysis software.

### Animal care

Murine melanoma studies were performed in a specific pathogen-free (SPF) breeding house in the animal facility of the Department of Experimental Pharmacology, National Institute of Oncology (Budapest, Hungary). The animals used in these studies were cared for according to the “Guiding Principles for the Care and Use of Animals” (Guide for the Care and Use of Laboratory Animals) based upon the Declaration of Helsinki, and approval was obtained from the local ethics committee (Ethics Committee of the National Institute of Oncology (Budapest, Hungary). The animals were fed a sterilized standard diet (Agroster, Budapest, Hungary) and had free access to tap water. They were kept in Makrolon cages at 23–25 °C (40–50% humidity), with a 12/12 h light/dark regimen. In all of the experiments, 8–10-week-old mice weighing 22–24 g were used.

### In vivo study

B16F10 murine melanoma cells were maintained in a humidified atmosphere of 5% CO_2_ at 37 °C and cultured in DMEM (Sigma) supplemented with 10% FBS (Sigma) and 100 µg/ml penicillin–streptomycin. 6.5 × 10^5^ cells were inoculated subcutaneously into the intrascapular region of 8–10-week-old C57BL/6 black mice. The mice were randomized, and treatment was started after the tumors reached a measurable size. The dosage of FWGE was 1432 mg/kg daily as determined in previous in vivo studies, which we do not describe in this article. The dosage of A250 was 43 mg/kg daily, which was calculated from its relative quantity in FWGE as determined from the yield of the isolation method. Both samples were dissolved in PBS, and up to 250 μL of solution was administered by oral gavage once a day for 14 days. Tumor growth was evaluated based on twice-weekly measurement of tumor volume with a digital caliper. The tumor samples were collected and snap-frozen in liquid nitrogen for protein analysis or formalin-fixed for hematoxylin and eosin (H&E) staining.

### Tissue sample collection

Blood was collected by cardiac puncture using 23G needles. Next, the subcutaneous tumor and the liver were removed immediately, divided in half, and kept in labeled dishes containing physiological saline. One half of each sample was frozen in 2-methylbutane (Isopentane, Sigma) chilled in liquid nitrogen. After that, samples were kept in labeled bags at − 80 °C. The other half of each sample was fixed in 8% formalin, dehydrated in a graded series of ethanol, infiltrated with xylene, and embedded in paraffin at a temperature not exceeding 60 °C. Two-micron-thick sections were mounted on SuperFrost slides (Thermo Shandon, Runcorn, UK) and manually deparaffinized. H&E-stained slides were used for routine pathological examination.

### Blood collection and hematological examinations

Animals were anesthetized with ketamine/xylazine, and blood was collected from the left ventricle of the heart with 23G needles into heparinized tubes. Plasma was separated by centrifugation. Then, 130 μL of whole blood was placed in a hematology machine (SysMex, XE 2100), and 150 μL of plasma was used for the measurement of liver enzymes (Olympus AU-640).

### Flow cytometry

For flow cytometry, 2 μL of the mixture of 2–2 antibodies was added to 50 μL of whole blood in a tube. Anti-Mouse CD4-PE (BD Pharmingen, 553048) / Anti-Mouse CD 19-FITC (eBioscience, 11-0193-82) or Anti-Mouse CD8a-PE (BD Pharmingen, 553032) / Anti-Mouse F4/80-Alexa Fluor 647 (Caltag, MF48021) were used together. Samples were incubated for 30 min in the dark at room temperature (RT). Then, 1 mL of 1 × eBioscience 1-step Fix/Lyse solution (eBioscience, 00-5333-57 10 × Solution) was added to the samples and vortexed gently. Samples were incubated for 30 min in the dark at room temperature. After a washing step (centrifugation at 500 × g for 5 min, wash once with 1 ml flow stain buffer, and spin again), cells were resuspended in 200 μL flow stain buffer. Samples were analyzed by a flow cytofluorometer (BD FACSCalibur). Data were analyzed using FlowJo software: FSC/SSC panel: R1 gate: living cells; FL1/FL2 panel: CD19-FITC/CD4-PE, CD19- and CD4-positive cells in R1; FL2/FL4 panel: CD8-PE/F4/80-A647, CD8- and F4/80-positive cells in R1.

## Results

### Fraction A250, the pool of active compounds of FWGE

**Figure 1:**
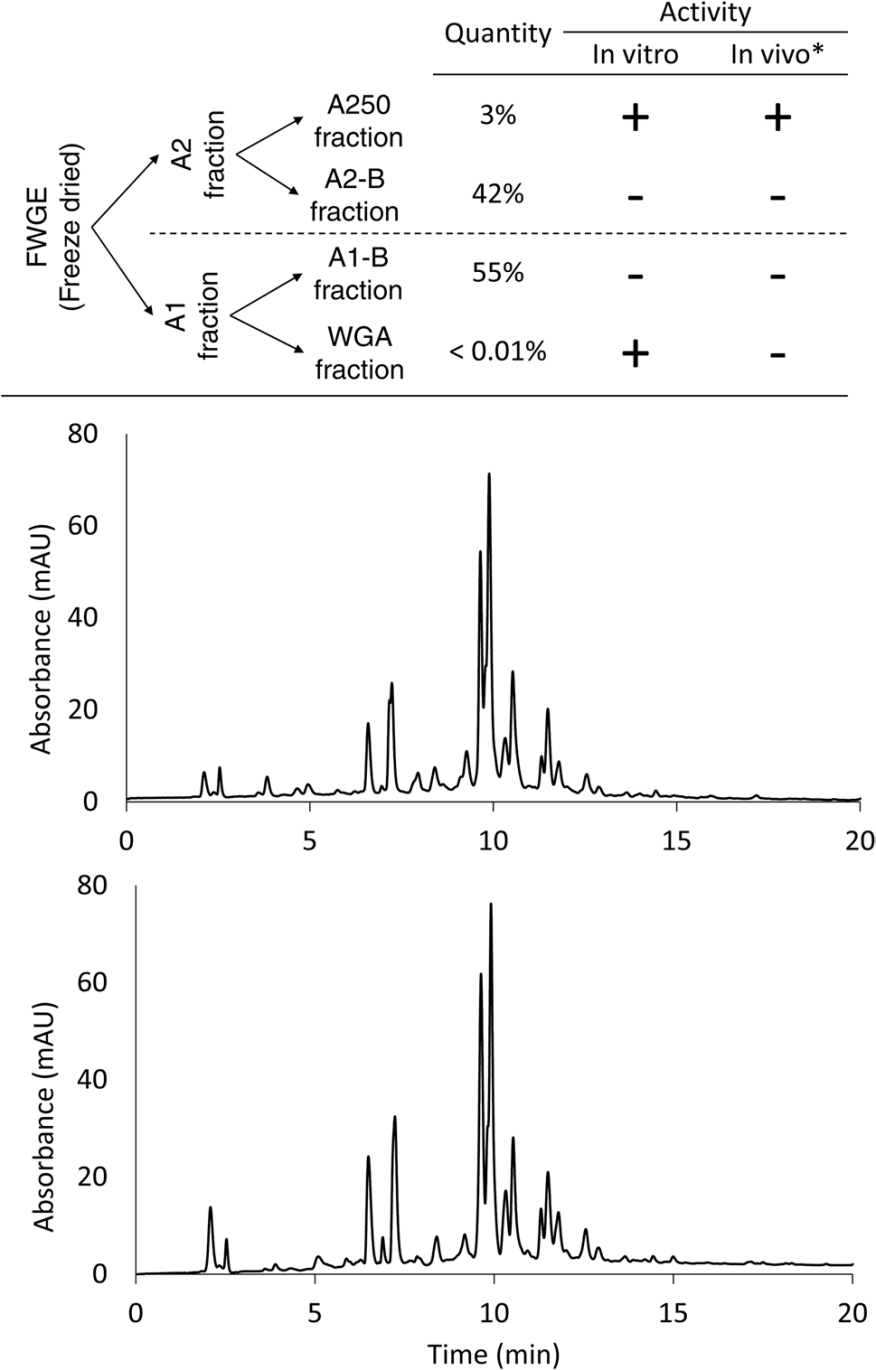
Fractionation flowchart. Whole FWGE was divided into two fractions with alcoholic extraction. WGA was the only in vitro active component of the alcohol insoluble part (**A1**), while the in vivo active components of FWGE were isolated from the alcohol-soluble part (**A2**) with SPE (Fraction A250). To validate the method, extraction was performed from two batches of whole FWGE. One was made in Europe, and the other was made in the US. Their HPLC fingerprint chromatograms (**B** and **C**) are nearly identical, showing not only that the method is reproducible but also that the active fractions can be isolated from different sources of wheat germ and baker’s yeast. The chromatograms were recorded in Thermo Xcalibur (version 3.0) software.

Freeze-dried FWGE was divided into two subfractions by resuspending in methanol. Fraction A2 represented about 45% of whole FWGE, and the remaining 55%, called Fraction A1, was insoluble in the organic solvent. Although both fractions were found to inhibit proliferation in vitro, Fraction A1 significantly lost its activity after wheat germ agglutinin (WGA) was removed with affinity chromatography using immobilized chitin antibody. The active components of Fraction A2 were concentrated into a subfraction with SPE. This subfraction, called Fraction A250, represented 3% of whole FWGE. Because Fraction A2-B did not show significant antiproliferative activity, Fraction A250 contained all of the in vitro active components of whole FWGE other than WGA (Fig. 1A).

To validate the isolation method of Fraction A250, we used different batches of whole FWGE and found that all batches showed a Fraction A250 quantity of around 3%, and all of these showed similar antiproliferative activity on the test cell lines. Two batches of A250 were compared with analytical methods, and the fingerprint chromatograms indicated that the chemical compositions were nearly identical. The intensities and retention times of the most abundant components were similar in the samples made from FWGE batches produced in either the US or Europe using different sources of wheat germ and baker’s yeast (Figs. 1B,C).

### Differential cellular sensitivity to A250 in vitro

**Figure 2:**
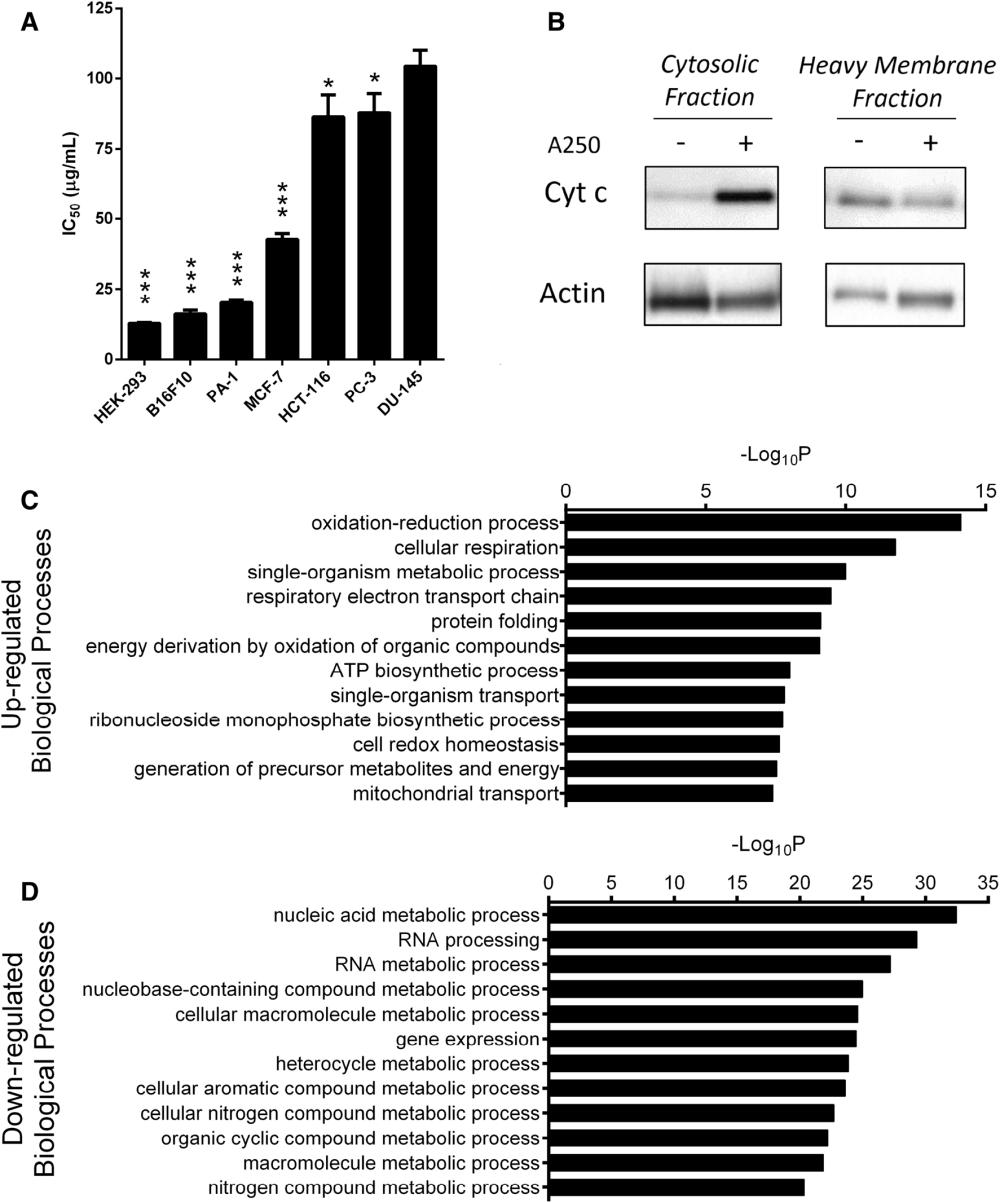
In vitro results of A250 treatment. (**A**) The efficacy of A250 was screened on different cancer cell lines. The mean IC_50_ was calculated from at least three measurements (Mean ± SD, one-way ANOVA, *p < 0.05, ***p < 0.001). (**B**) Mitochondrial-derived apoptosis was validated with an immunoblot assay. The significant increase of cytochrome c in the cytosolic fraction indicates that the mitochondrial outer membrane permeability increased after treatment. β-actin was used as the loading control. (C,D) GO interpretation of the iTRAQ results of the in vitro-treated melanoma cells shows significant upregulation of metabolic and respiratory processes. In contrast, proteins related to RNA metabolic processes and gene expression were downregulated by the treatment with A250. (The unedited pictures of SDS-gel electrophoresis and immunoblot can be found in the *Supplementary information*. SI—Fig. 1–3).

The antiproliferative activity of Fraction A250 was screened on different immortalized or cancerous cell lines, such as HEK-293 T, B16F10, PA-1, MCF-7, HCT-116, PC-3, and DU-145, to select the most sensitive cells for further mechanistic investigation. The IC_50_ on the most sensitive cell line (HEK-293 T) was 12 μg/mL, while the most resistant cell line (DU-145) required an approximately tenfold higher concentration to reach a similar proliferation inhibition. This difference in efficacy indicated that some cell lines were more sensitive to the active compounds of A250 (Fig. 2A).

### Cytochrome c release indicates activated intrinsic apoptosis

We used the immunoblotting technique to detect the activation of mitochondria-mediated apoptosis after A250 treatment. One of the most sensitive cell lines, HEK-293 T, was treated for 4 h and lysed into two subcellular fractions by using digitonin in the first step and NP-40 in the second step. After 4 h of treatment, the level of cytochrome c was highly increased in the cytosolic fraction and reduced in the heavy membrane fraction, indicating increased permeability of the mitochondrial outer membrane and release of cytochrome c into the cytosol (Fig. 2B).

### Increased mitochondrial protein levels

We conducted quantitative proteomic analysis by lysing, digesting, and labeling the proteins of treated and control cells to determine the effect of A250 on gene expression levels. We used B16F10 murine melanoma cells—the second most sensitive cell line in our prior screen—because we needed a cell line that could be used to validate the in vivo effect as well. More than 4000 proteins were detected and identified in this proteomic analysis. Biological roles and relationships among the most significantly up- or down-regulated proteins were analyzed with data analysis methods. Based on the GO Term Biological Process analysis, oxidation–reduction processes and cellular respiration were represented by the significantly upregulated proteins. Furthermore, most of the upregulated proteins were located in the mitochondria or the cytosol but were involved in glucose metabolism and energy production (Fig. 2C). Besides the effect on respiration, we also found that A250 decreased the levels of proteins related to gene expression and RNA processing (Fig. 2D).

### Mitochondrial respiration assay

**Figure 3:**
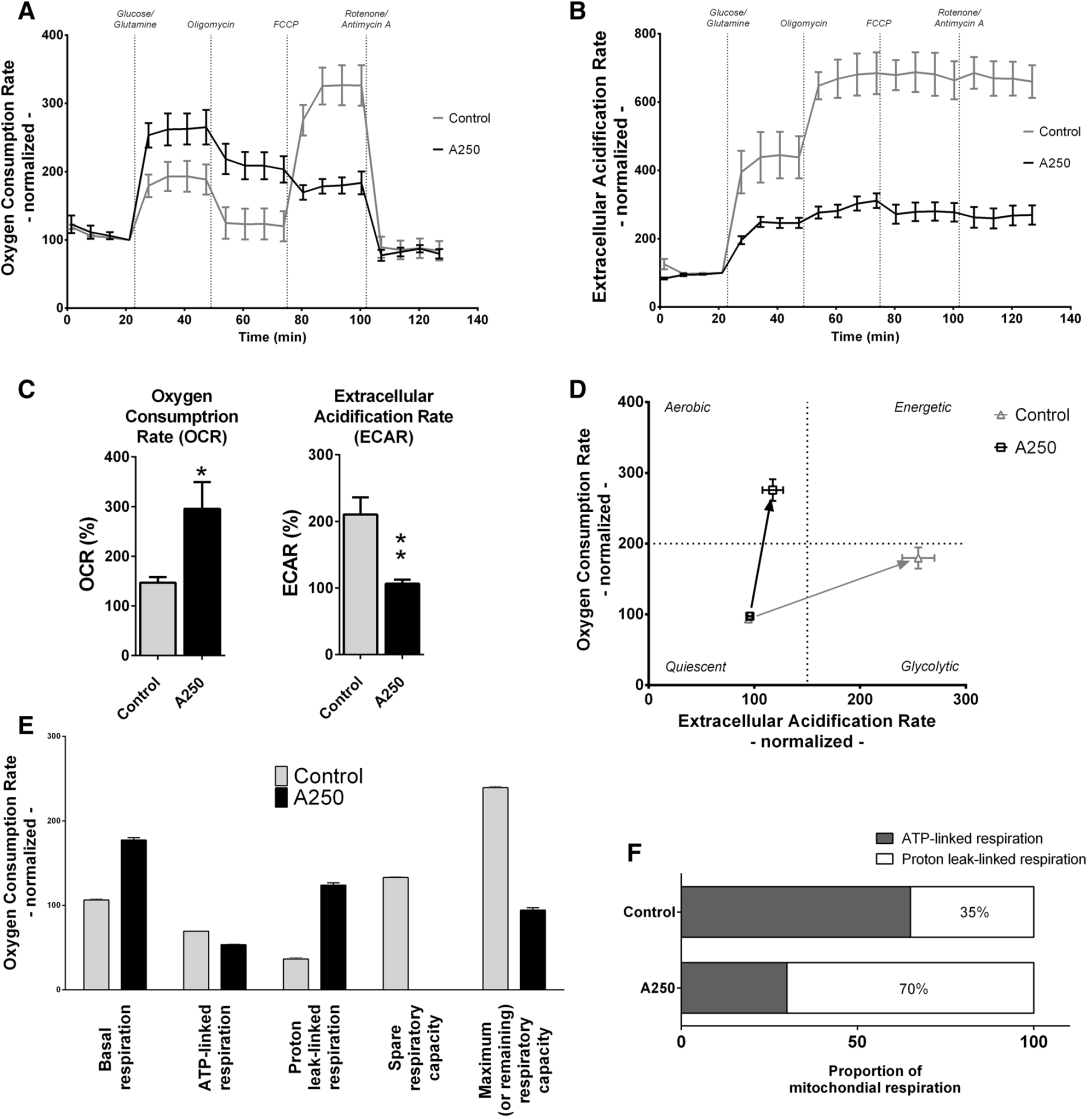
Oxygen consumption and extracellular acidification. The OCR and ECAR were monitored to validate the impact on respiration and glycolysis. B16F10 melanoma cells were treated with A250 in glucose- and glutamine-free medium. (**A**) The OCR curve of the treated cells is significantly different from that of the non-treated cells. (Mean ± SEM) The higher level of OCR indicates more active respiration. (**B**) The lower level of ECAR, on the other hand, indicates a lower quantity of lactic acid, which represents reduced glycolytic activity. (**C**) After injection of glucose and glutamine, we measured an approximately two-fold higher OCR and about two-fold lower ECAR. Both results are significant (Mean ± SEM, one-way ANOVA, *p < 0.05, **p < 0.01). (**D**) The energy phenotype profile (EPP) of the treated cells changed from quiescent to aerobic in the presence of glucose and glutamine, while the EPP of the non-treated cells changed to glycolytic in the same condition. The difference in EPP indicates that the cancer-specific cellular metabolic phenotype (Warburg effect) was less active, and normal metabolic activity was more prominent in the treated cells. (Mean ± SEM) (**E**) Different stages of cellular respiration can be determined from the OCR curve. Spare respiratory capacity is the measure of OCR in the presence of FCCP. The lack of spare respiratory capacity in the treated cells needs to be investigated. (**F**) The analysis of mitochondrial respiration shows that proton leak-linked respiration was responsible for 70% of the OCR, compared to only 35% in the control cells. Graphpad Prism 5.02 software was used for data analysis.

Next, we used the Seahorse Extracellular Flux Analyzer to measure the mitochondrial respiration and glycolytic activity of B16F10 melanoma cells^30^. Cells were treated with A250 overnight, and then flux analysis was performed. Although the basal OCR was unchanged in the absence of nutrient substrates, the OCR increased to a greater extent in the A250-treated cells following injection of glucose and glutamine (Fig. 3). Although the OCR remained higher after injection of the ATP synthase inhibitor oligomycin, the similar drop in OCR indicates that the treatment did not affect ATP-linked respiration but rather caused a significant increase in the proton leak-linked aspect of mitochondrial respiration. Whether the inducible or passive form of proton leak becomes more active in this context requires further investigated. Interestingly, the uncoupler FCCP did not increase the OCR to the maximum respiratory capacity of the treated cells. Non-mitochondrial respiration remained the same, as the administration of rotenone and antimycin-A—two molecules that completely shut down the electron transport chain—reduced the OCR to the same level as in the control cells.

Besides the effect on the OCR, A250 treatment reduced glycolysis-related lactic acid production. The ECAR was significantly lower in the presence of glucose due to pyruvate being transferred into the mitochondria rather than being utilized in lactic acid production and released into the extracellular space. Lactic acid production remained low even after ATP production was inhibited by oligomycin.

Overall, these data demonstrate that A250 increases mitochondrial activity, as evidenced by the increase in OCR, whereas the decrease in ECAR suggests reduced glycolytic flux.

### Metabolomics study

**Figure 4:**
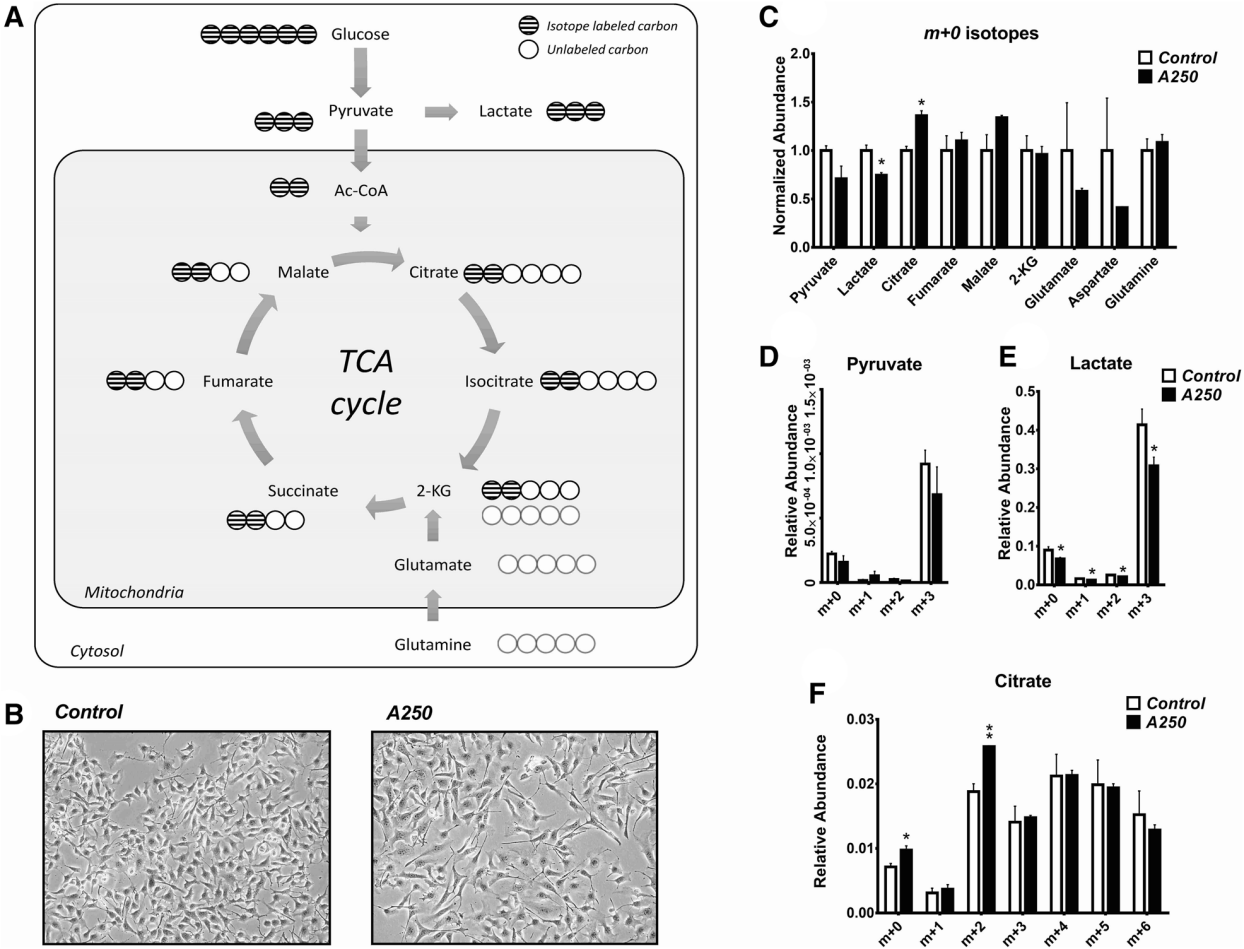
Metabolomics results. The effect of A250 on the cancer-specific cell metabolism was validated in B16F10 melanoma cells using isotope-labeled glucose. (**A**) [U6-^13^C_6_]-glucose is converted to pyruvate in glycolysis. It can be either transported into the mitochondria and converted to Acetyl-CoA, or converted into lactic acid while still in the cytosol. Either way, all carbons of pyruvate and lactic acid are derived from the isotope-labeled glucose. (**B**) The treatment reduced the proliferation rate and affected the morphology of B16F10 cells after 24 h incubation. (**C**) The *m* + *0* isotopes of the main substrates of the TCA cycle were quantified with GC–MS. The abundance was normalized to the control sample. We detected a significant decrease in lactic acid quantity and a significant increase in citrate quantity. (**D**) The *m* + *3* isotope of pyruvate was the most abundant, and the treatment did not change the pyruvate level significantly. (**E**) The *m* + *3* isotope of lactic acid was the most abundant, but in this case, the treatment significantly decreased its production. (**F**) All seven isotopomers of citrate were monitored, and the very significant increase of the *m* + *2* isotope demonstrates the transport of pyruvate into the mitochondria rather than its conversion into lactic acid (one-way ANOVA, *p < 0.05, **p < 0.01). *Figure 4A* was drawn by GB. Graphpad Prism 5.02 software was used for data analysis.

Next, we performed a ^13^C stable tracer analysis on B16F10 murine melanoma cells. We analyzed isotopomers for metabolites of glycolysis and the tricarboxylic acid (TCA) cycle and found that in the A250-treated melanoma cells, the total lactic acid level and especially m + 3 lactate were significantly lower compared to the control cells. The increased quantity of m + 2 citrate in A250-treated cells further demonstrated increased flux into the TCA cycle and oxidative metabolism (Fig. 4).

Because we used isotope-labeled glucose in the media, the m + 3 isotope of lactic acid and m + 2 isotope of citrate were the dominant isotopes in the samples. Interestingly, there did not appear to be a change in pyruvate, although there was a trend toward reduced m + 3 pyruvate. These data demonstrate that A250 promotes the entry of glucose-derived carbon into the TCA cycle and away from lactic acid and the classic Warburg effect.

### In vivo efficacy study

**Figure 5:**
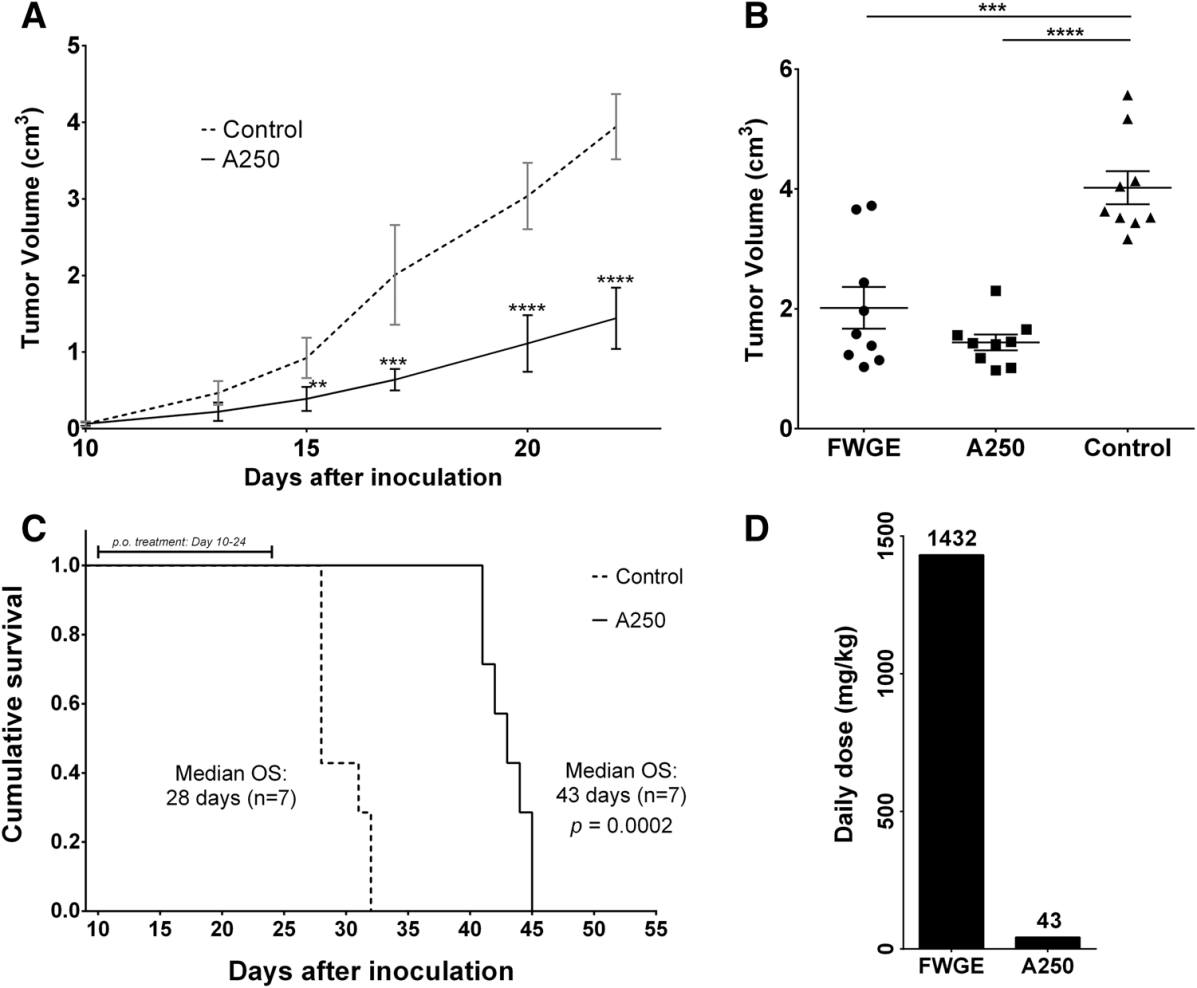
In vivo tumor growth results. Comparison study in the subcutaneously injected mice melanoma model. The treatment was started on the 10^th^ day after inoculation. (**A**) The tumor growth curves show a significant difference between the control and treated groups from the fifth day of treatment (Mean ± SD). (**B**) At the end of the study, the mean tumor volume in the treated groups was more than 50% less than that of the control group. There was no significant difference between the efficacy of whole FWGE and A250 (Mean ± SEM). (**C**) The Kaplan–Meier survival curve shows a more than 50% increase in median OS in the treated group. (**D**) The daily dose of A250 was calculated from the isolation yield, and thus it was administered at a 33-fold lower dose than that of whole FWGE (one-way ANOVA, **p < 0.05, ***p < 0.001, ****p < 0.0001). Graphpad Prism 5.02 software was used for data analysis.

The in vivo activity of A250 and the original extract were compared in the murine melanoma model of subcutaneously injected B16F10 cells. The mice were randomized once the mean size of the tumors reached approximately 0.06 cm^3^. The tumor growth curve shows that treatment with A250 significantly inhibited tumor growth (Fig. 5A).

After 14 days of treatment, FWGE and A250 both significantly reduced tumor size. The mean tumor size in the control group was 4.02 cm^3^. By this time, the mean tumor size in the group treated with whole FWGE was around 2 cm^3^ (2.02 cm^3^, *p* < 0.0001, n = 7) compared to less than 2 cm^3^ in the group that was treated with A250 (1.44 cm^3^, *p* < 0.0001, n = 7) (Fig. 5B).

When we measured the overall survival (OS) of the animals treated with the previously described dosages, we found that the median OS of the control animals was 28 days, counted from tumor inoculation, while the median OS in the treated group was 43 days (*p* = 0.0002) (Fig. 5C).

### Impact on gene expression in vivo

**Figure 6:**
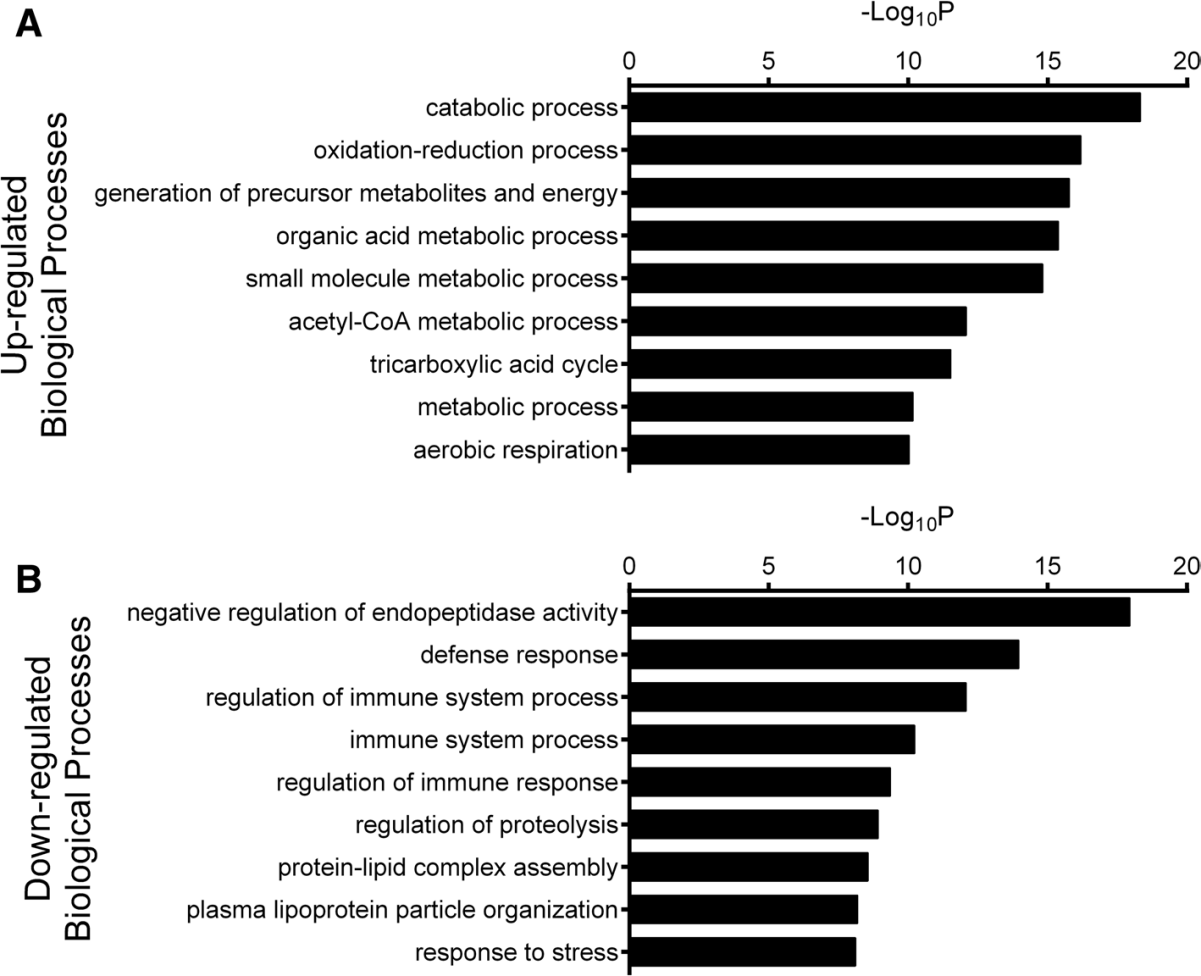
In vivo iTRAQ results (GO-Biological Processes). (**A**) GO interpretation of the iTRAQ results of the in vivo-treated melanoma cells shows significant upregulation of metabolic, TCA cycle, and respiratory processes. (**B**) In contrast, processes involved in endopeptidase activity and regulation of the immune system were downregulated upon treatment with A250. The iTRAQ data was analyzed on STRING Potein-protein Interaction Network website (Version 9.0). (https://string-db.org/).

Tumor samples from the in vivo study were collected after 14 days of treatment. Samples were processed and analyzed as described in the in vitro section. Similar to the in vitro proteomic studies, mitochondrial and metabolic pathways were represented by many of the upregulated proteins (SI-Fig. 4A,B). Interestingly, for the in vivo samples, various catabolic pathways were some of the most significantly affected biological processes (Fig. 6).

In contrast to the similarity of the pathways upregulated in vitro and in vivo, the significantly downregulated proteins in vivo differed from those in vitro. The most downregulated biological processes were associated with endopeptidase activity, the defense response, and regulation of the immune system. Stress response proteins were also less abundant in the treated tumor samples (SI-Fig. 4C,D).

### Effect on liver tissue and blood count

After two weeks of A250 treatment, livers were collected and analyzed with H&E staining. The lack of structural differences in liver tissue of the treated and control animals indicated that the test compound did not have general toxic properties (SI-Fig. 5).

In addition, mice were monitored for weight during the study. Although all of the animals gained a significant amount of body weight, the difference between the treated and control groups was not statistically significant (SI-Fig. 6).

Complete blood count (CBC) results in the treated and untreated groups were very similar, except for red blood cell (RBC) and platelet (PLT) numbers and the percentage of CD4 + and CD8 + T cells (SI-Table 1). For each of these four metrics, the treated tumor-bearing mice had significantly higher counts than the non-treated mice, approaching counts in healthy mice. The mean RBC number in the treated group was 4.27 × 10^6^ µL^−1^ vs. 2.86 × 10^6^ µL^−1^ in the control group (*p* = 0.0382)*.* The mean PLT number was 230 × 10^3^ µL^−1^ vs. 147 × 10^3^ µL^−1^ in the control group (*p* = 0.0397)*.* The mean quantity of CD4 + T cells was 10.8% vs. 8.26% in the control group (*p* = 0.0211), and the mean quantity of CD8 + T cells was 7.89% vs. 6.35% in the control group (*p* = 0.0091). Similar to most of the CBC results, the differences in liver enzyme levels were not statistically significant between the treated and control groups, indicating that the treatment itself had no toxic effect on liver tissue.

## Discussion

Standardized FWGE is a complex mixture of molecules made by the fermentation of wheat germ by baker’s yeast and has been the subject of extensive study in cell lines, animals, and in clinical trials, showing remarkable anticancer effects and safety. We concentrated the active components through bioassay-guided fractionation and found that alcoholic extraction, followed by SPE, could isolate the molecules with in vivo activity into a highly reproducible fraction, called A250, which represents approximately 3% of whole FWGE.

The activity of A250 was tested in cell proliferation assays on seven different cancer cell lines, which showed different sensitivity. The IC_50_ of the most sensitive HEK-293 T cell line was about tenfold lower than that of the least sensitive prostate cell line. The apoptosis-inducing activity of A250 was validated with an immunoblot assay by probing the samples with an antibody against cytochrome c. The fractionated lysing of the treated cells revealed increased levels of cytochrome c in the cytosolic fraction with a slight decrease in the heavy membrane fraction. As cytochrome c is an essential member of the mitochondrial apoptotic pathway, its presence in the cytosol is a marker of mitochondria-induced apoptosis^31,32^.

Using proteomic analysis, we detected upregulated expression levels of several mitochondrial proteins. Most of these proteins are related to normal cellular metabolism, while others are involved in protein folding and transport. The downregulated proteins were ribosomal- and gene expression-related proteins. These results indicate that A250 has a significant impact on the cellular energy balance through mitochondria, which also explains the reduced level of proliferation-related proteins as a consequence of nutrient deprivation.

We validated the impact on the mitochondria using a Seahorse Bioanalyzer. Both the increased OCR and decreased ECAR—indicating reduced glycolysis resulting from decreased lactic acid production—show that A250 treatment inhibits the Warburg effect and increases the metabolic activity of the mitochondria. The increased carbon flux to the mitochondria was validated using stable isotope tracer analysis. A250 treatment significantly decreased the lactic acid abundance and increased the levels of citric acid, especially that derived from glucose, the first metabolic substrate of the TCA cycle. The pyruvate level was not significantly different between the treated and control cells.

We demonstrated that A250 is bioequivalent to whole FWGE in an in vivo murine melanoma xenograft model, even though it represents only 3% of the biomass of the original extract. In this study, we found that a 33-fold lower dosage of Fraction A250 inhibited the growth of subcutaneously injected B16F10 cells to the same degree as whole FWGE. During the 14-day-long treatment, Fraction A250 inhibited tumor growth by 68%. In addition, the survival of the mice was increased significantly from 28 to 43 days with only 14 daily dosages, representing an increase of more than 50%.

Proteomic analysis of the in vitro and tumor samples showed a similar increase in the level of mitochondrial proteins. This also shows that the active compounds of A250 are orally available and that they have a similar effect on the metabolic activity of cancer cells after being absorbed from the gastrointestinal tract. Interestingly, the significantly downregulated proteins were involved in autophagy and the defense response, which differed from the in vitro results and may represent a secondary effect in vivo. This finding requires further investigation.

Neither the CBC results nor the liver enzyme panel showed any signs of overall toxicity. The daily behavior of treated mice did not differ from that of the control group, and their body weight was normal during the treatment study. The H&E results also confirmed that the treatment did not cause any morphological changes in the liver. Necrotic activity was not reported.

## Conclusion

Here we demonstrate that Fraction A250, a highly concentrated form of the active compounds of FWGE, changes the acidic environment by reducing lactic acid production and reprograms cellular metabolism away from the Warburg effect to increase mitochondrial carbon flux and oxidation. We also show that A250 increased the permeability of the mitochondrial outer membrane, leading to the release of cytochrome c into the cytosol. Cytochrome c has a significant role in intrinsic, mitochondria-mediated apoptosis, and it amplifies apoptotic signals. The increased level of TCA cycle metabolites is expected to have further beneficial inhibitory effects on the glycolytic enzymes; therefore, in addition to initiating apoptosis through mitochondria, A250 has an active role in nutrient deprivation in cancer cells. As our results indicate, the effect of A250 on mitochondrial function reduces tumor growth in vivo and extends OS. In the 14-day treatment, the mice did not show any signs of toxicity, and pathological examination of the liver tissue validated that A250 had no toxic effect on normal tissue.

Cancer has been considered as Mitochondriopathy. Therefore, restoration of mitochondrial biology in a way that activates the Krebs’ cycle and manage apoptotic machinery might result in a breakthrough in the field of cancer medicine. One of those approach is that Schwartz et al., suggested the usage of metabolic treatment (Hydroxycitrate + α-Lipoic Acid) will restore the mitochondrial function^8,33,34^. Parallel of the context, the current work shows that FWGE has comparable pharmacological effect. As a non-toxic, orally available, and efficient in vivo tumor growth inhibitor, A250 has the potential to support standard chemotherapy treatments and targeted therapies by modulating cellular energy balance and enhancing apoptosis.

## Supplementary information

Supplementary Information 1.
