# Author Correction: Mito-oncology agent: fermented extract suppresses the Warburg effect, restores oxidative mitochondrial activity, and inhibits in vivo tumor growth

**DOI:** 10.1038/s41598-021-82411-0

**Published:** 2021-01-29

**Authors:** Gyula Bencze, Szilvia Bencze, Keith D. Rivera, James D. Watson, Mate Hidvegi, Laszlo Orfi, Nicholas K. Tonks, Darryl J. Pappin

**Affiliations:** 1 Cold Spring Harbor Laboratory, Cold Spring Harbor, NY 11724 USA; 2 Department of Pharmaceutical Chemistry, Semmelweis University, Budapest, 1085 Hungary; 3 Jewish Theological Seminary—University of Jewish Studies, Budapest, 1084 Hungary; 4 American Biosciences, Inc, Blauvelt, NY 10913 USA

Correction to: *Scientific Reports* 10.1038/s41598-020-71118-3, published online 25 August 2020

This Article contains errors.

Mate Hidvegi was omitted from the author list in the original version of this Article.

As a result, the Acknowledgements section,

“We are grateful to Mate Hidvegi for the generous gift of the freeze-dried fermented wheat germ extract used as the control in the in vivo study and as starting material in the isolation process. We are also thankful to Geoffrey Girnun and Emily Montal for their expertise in metabolomics and Seahorse analysis and for the helpful discussions.”

now reads:

“We are thankful to Geoffrey Girnun and Emily Montal for their expertise in metabolomics and Seahorse analysis and for the helpful discussions.”

The Author Contributions section now reads:

G.B., S.B. and K.R. carried out the experiment. G.B. wrote the manuscript with support from S.B., J.D.W, L.O., N.K.T and D.P. K.R. fabricated the proteomics sample. J.D.W and N.K.T. helped supervise the project. H.M. is the originator of the research and provided the freeze-dried form of fermented wheat germ extract. G.B. and D.P. conceived the original idea. D.P. supervised the project.

In addition, the authors neglected to include some potential conflicts of interest. The Competing Interests section now reads:

G.B. is an employee of American Biosciences Inc., and co-founder of Mitomarin Inc. G.B performed this research while he was a Visiting Scientist at Cold Spring Harbor Laboratory with financial support from American Biosciences Inc. G.B., L.O., D.P., H.M., N.K.T., and J.W. are inventors of patent applications covering A250 (Application number: 13/135,616 and 14/121,822, both now abandoned) H.M. has received travel compensation funds from American Biosciences, Inc. between 2009-2012. The rest of the authors declare no competing interests.

Lastly, an affiliation for author Gyula Bencze was omitted. The correct affiliations for Gyula Bencze are listed below:

Cold Spring Harbor Laboratory, Cold Spring Harbor, NY, 11724, USA

American Biosciences, Inc, Blauvelt, NY 10913, USA

These errors have now been corrected in the PDF and HTML versions of the Article.

