# Supplementary material for: Mito-oncology agent: fermented extract suppresses the Warburg effect, restores oxidative mitochondrial activity, and inhibits in vivo tumor growth
Source: Sci Rep. 2020 Aug 25;10:14174. doi: 10.1038/s41598-020-71118-3 (PMC7447799; doi:10.1038/s41598-020-71118-3)
Supplement: Supplementary file 1 — Supplementary Information 1. [file 41598_2020_71118_MOESM1_ESM.docx]

***Supplemental information:***

**SI – Figure 1:** SDS-Gel Electrophoresis of cytosolic and heavy membrane fraction of the cells. (A) Cytosolic fraction of the treated HEK-293-T cells. (B) Cytosolic fraction of the non-treated HEK-293T cells. (C) Heavy membrane fraction of the treated HEK-293T cells. (D) Heavy membrane fraction of the non-treated HEK-293T cells. The protein quantity of the sample was determined with Bradford reagent and the same quantity was loaded in each well. BIORAD Kaleidoscope protein ladder was loaded in well No. 1 and No. 10

**SI – Figure 2:** Western blot with Anti-Cytochrome c antibody

Cytochrome c level in the cytosolic fraction (A) and he heavy membrane fraction (B) of the treated and non-treated HEK-293T cells, respectively.

**SI – Figure 3:** Western blot with Anti-Actin antibody

Actin level in the cytosolic fraction (A) and he heavy membrane fraction (B) of the treated and non-treated HEK-293T cells, respectively.

**SI – Figure 4:** *In vivo* iTRAQ figure

1. (A) STRING Protein association network of the significantly overexpressed proteins of the subcutaneously injected B16F10 murine melanoma cells treated with A250 for 14 days. Confidence view with a 0.900 required minimum score. The colored nodes show the proteins involved in metabolic pathways, those related to mitochondria, or both. (B) Zoomed-out view of the mitochondria-related cluster of 52 proteins. The nodes are labeled with the names of the proteins, and the different colors represent different metabolic functions. (C) STRING Protein association network of the significantly downregulated proteins of the subcutaneously injected B16F10 murine melanoma cells treated with A250 for 14 days. Confidence view with a 0.900 required minimum score. The colored nodes show the proteins involved in the regulation of proteolysis or the immune system and those involved in the defense system. (D) The nodes are labeled with the names of the proteins, and the different colors represent a role in either the regulation of proteolysis or the stress response. The iTRAQ data was analyzed on STRING Potein-protein Interaction Network website (Version 9.0). (https://string-db.org/)

**SI – Figure 5:** H&E results of tumor and liver tissue samples

Pathological examination of liver tissue from the control and treated animals did not show any treatment-specific morphological or structural changes in the liver.

**SI – Figure 6:** Body weight of mice during the treatment

Mouse body weight was measured every other day during the treatment. (A) The growth curves of the control and treated mice were linear. The animals did not lose any weight during the treatment, and they gained about 30% of their initial body weight by the end of the treatment (Mean ± SD). (B) Although the difference in body weight between the first and last day of the treatment was significant in both groups, the difference between the groups was not significant. (Median, Box = SD, Whiskers = Min/Max, **** p<0.0001)

**SI – Table 1:** CBC and liver panel table

A liver enzyme panel was performed to detect toxicity to normal tissues. Although the levels of three of four enzymes were elevated relative to the healthy mice, the levels in the non-treated tumor-bearing mice were not significantly different (GOT=Aspartate aminotransferase, GPT=Alanine aminotransferase, LDH=Lactate dehydrogenase, BIO= Biotinidase). A CBC was performed to determine the effect of A250 on blood cells. We detected a significant difference between the non-treated and treated mice in two measures. The RBC and PLT numbers were higher in treated cells; interestingly, these numbers were closer to normal levels, compared to the untreated control animals. No significant changes were detected between the control and treated groups in the Neutrophil (NEUT), Monocyte (MONO), or Lymphocyte (Lymph) counts. While the percentage of CD19+ B lymphocytes was not significantly different, the percentages of CD4+ and CD8+ T lymphocytes were found to be significantly higher in the treated mice relative to the control animals. As with the RBC and PLT numbers, these percentages were closer to normal levels compared to the control animals.


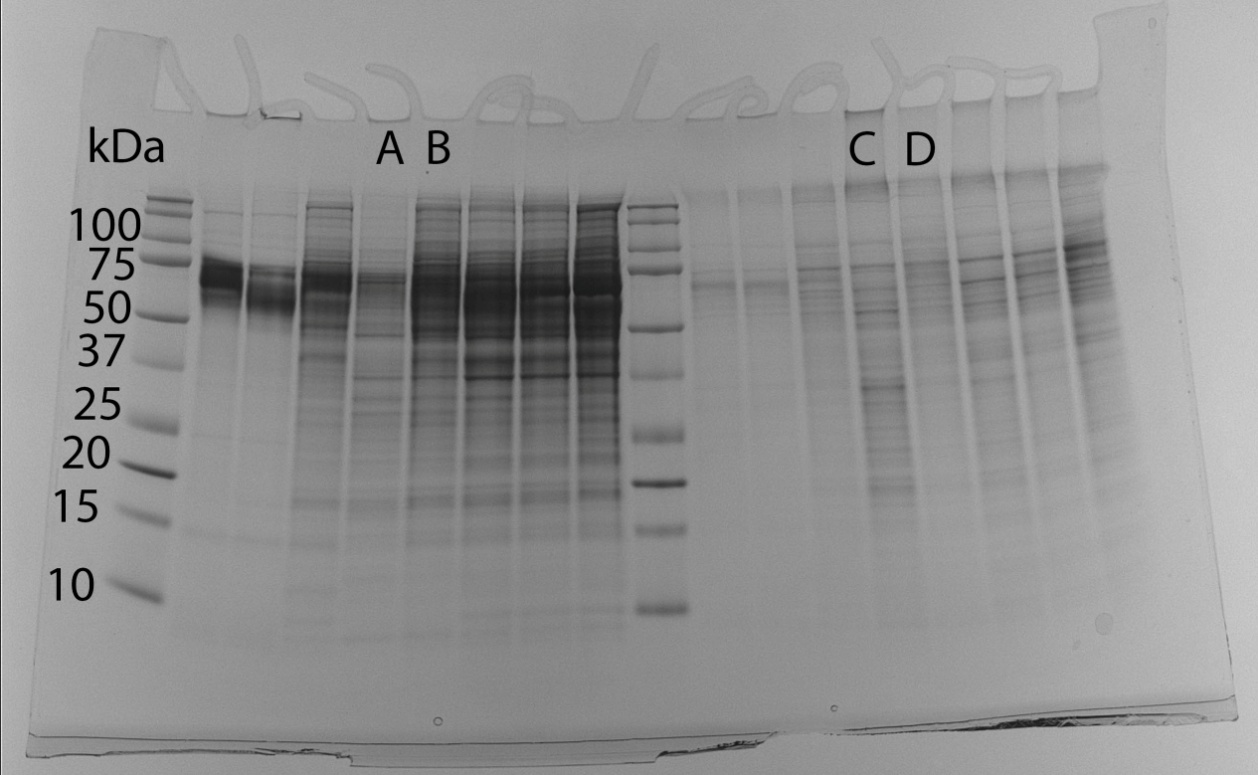


SI - Figure 1


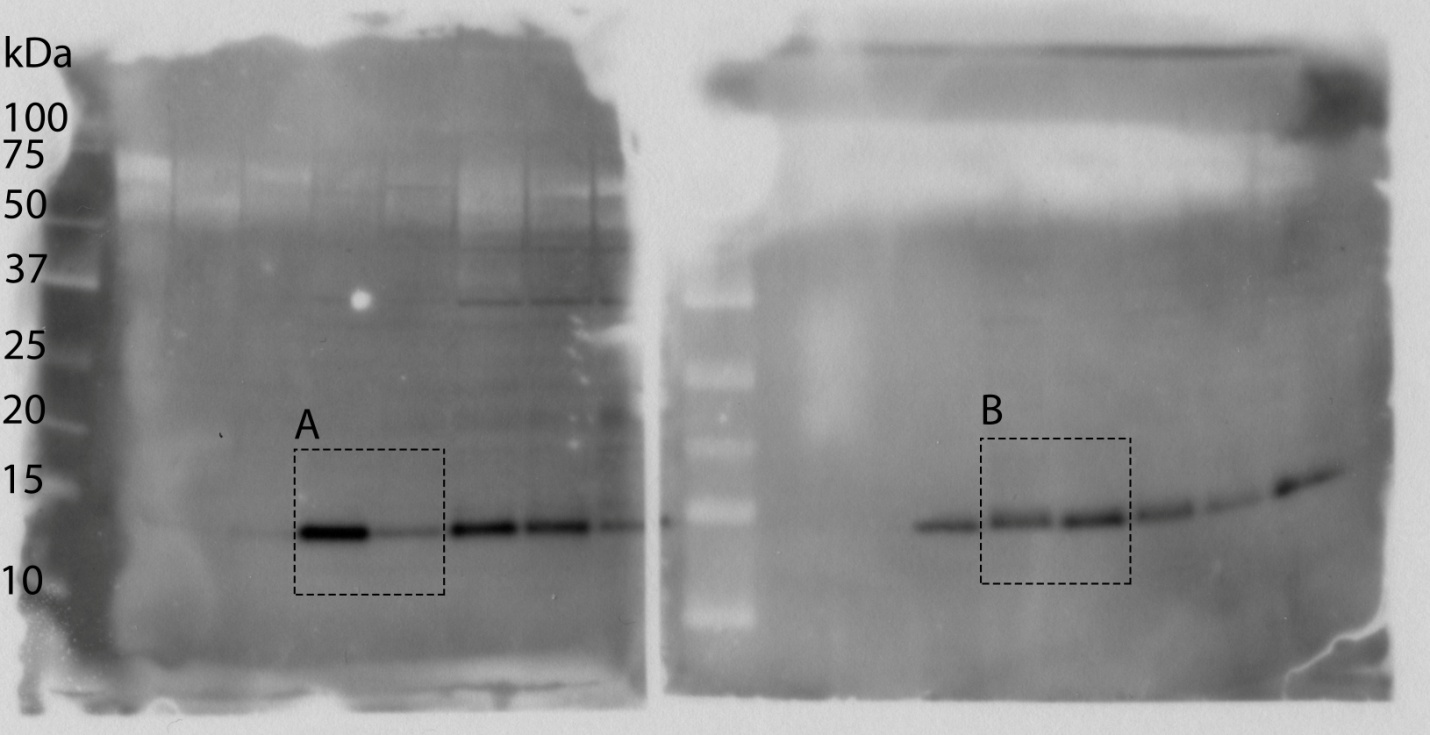


SI - Figure 2


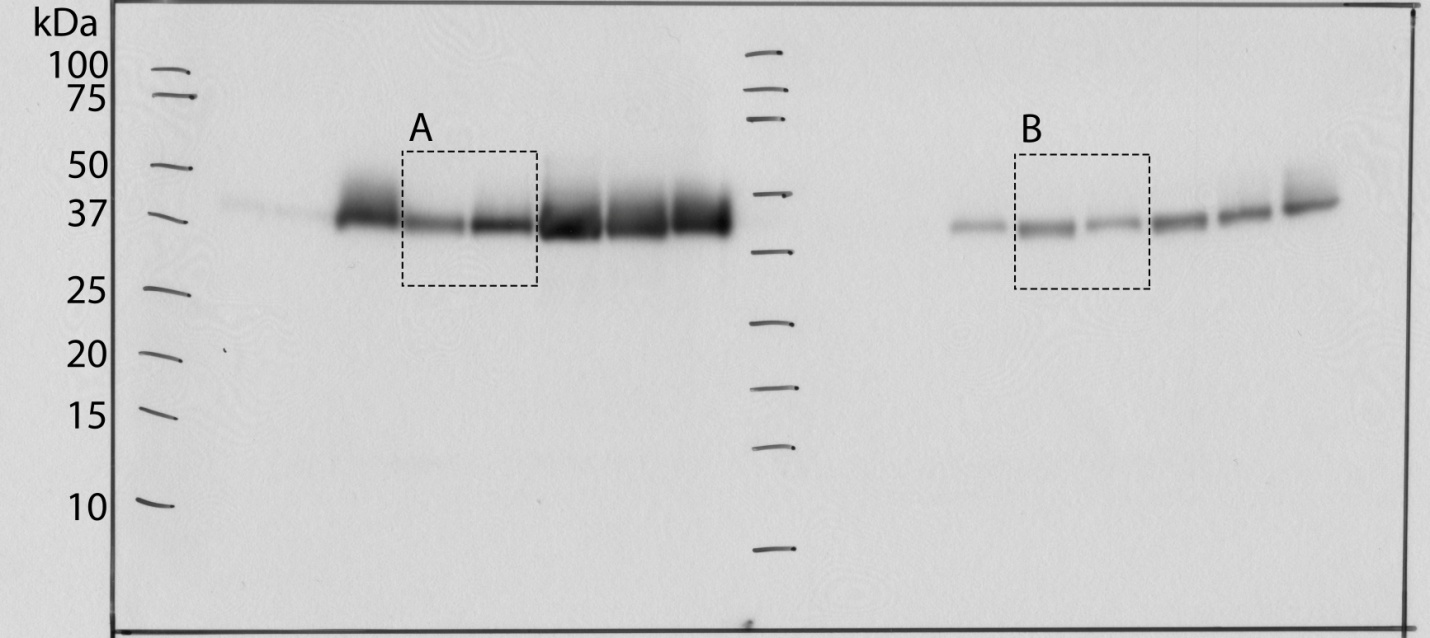


SI - Figure 3


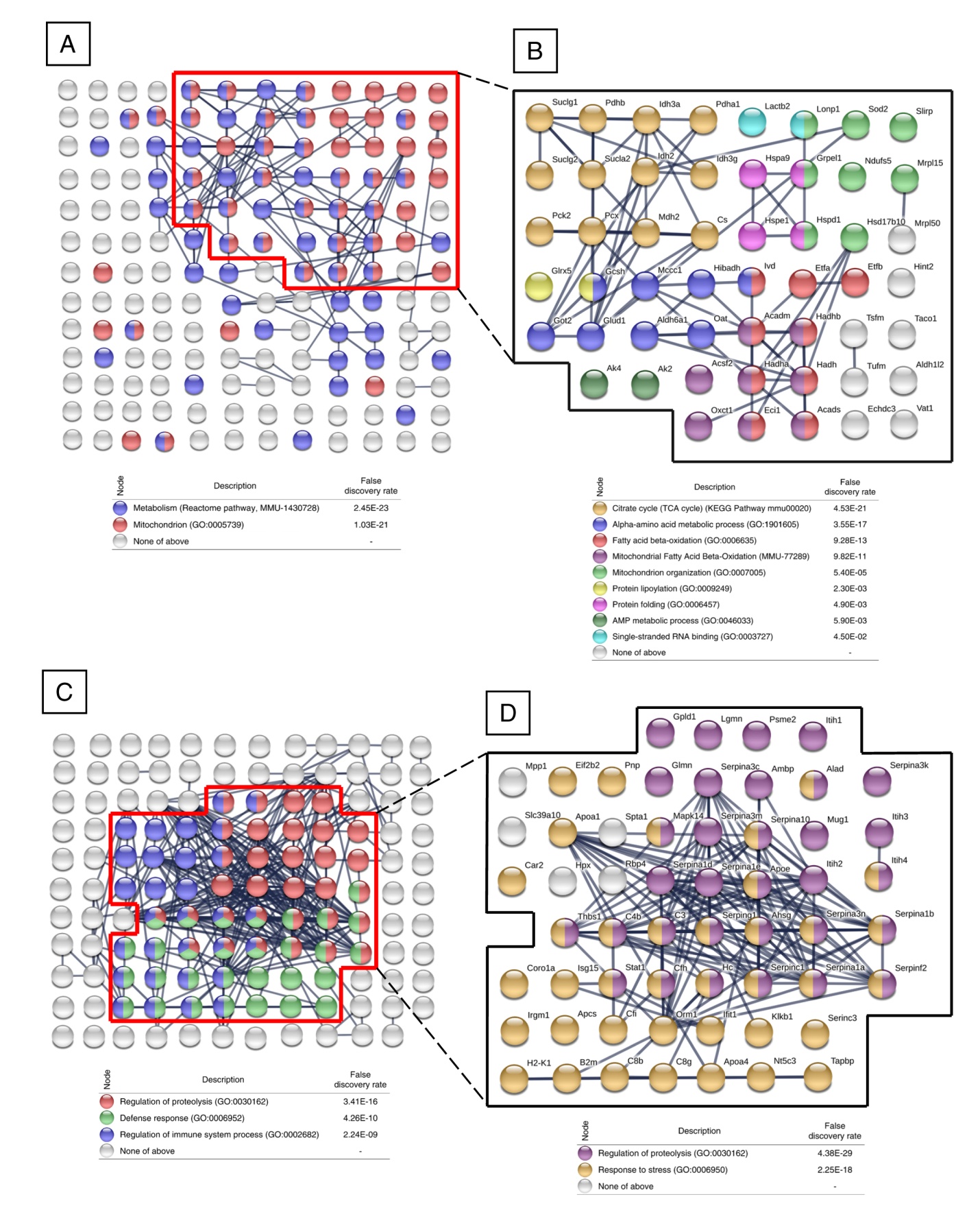


SI - Figure 4

***Liver from untreated, tumor-bearing mice (40x, 100x, 200x)***

***Liver from treated, tumor-bearing mice (40x, 100x, 200x)***

***Tumor from untreated group (40x, 100x, 200x)***

***Tumor from treated group (40x, 100x, 200x)***

SI - Figure 5

*

SI - Figure 6*

SI – Table 1. CBC and liver enzyme results of normal and tumor-bearing mice (with and without A250 treatment)

|  | | **Healthy mice** | **Tumor-bearing mice** | | ***p*-value** |
| --- | --- | --- | --- | --- | --- |
|  |  |  | **Non-treated** | **Treated** |  |
| **GOT** | | 50.5 ± 10.61 | 1560.67 ± 444.03 | 1333.33 ± 456.01 | 0.3571 |
| **GPT** | | 16.5 ± 19.09 | 90.83 ± 35.42 | 94.67 ± 36.02 | 0.8421 |
| **LDH** | | 311 ± 0 | 30671.4 ± 16846.7 | 20175.78 ± 12759.23 | 0.2666 |
| **BIO** | | 6.25 ± 6.29 | 8.85 ± 11.4 | 2.09 ± 1.42 | 0.2069 |
| **WBC (10³/µL)** | | 5.27 ± 0.7 | 5.17 ± 1.3 | 6.24 ± 1.5 | 0.1785 |
| **RBC (10^6^/µL)** | | 7.89 ± 0.23 | 2.86 ± 0.65 | 4.27 ± 1.5 | ***0.0382*** |
| **HGB (g/dL)** | | 11.6 ± 0.42 | 6.32 ± 4.32 | 6.69 ± 2.12 | 0.8521 |
| **HCT (%)** | | 37.8 ± 1.41 | 34.97 ± 39.84 | 24.95 ± 6.62 | 0.5677 |
| **PLT (10^3^/µL)** | | 434.5 ± 60.1 | 147.2 ± 47.64 | 230.38 ± 80.85 | ***0.0397*** |
| **NEUT** | **(10^3^/µL)** | 0.5 ± 0.11 | 1.83 ± 0.88 | 2.02 ± 0.44 | 0.7456 |
|  | **(%)** | 9.65 ± 3.32 | 33.23 ± 7.35 | 34.89 ± 7.9 | 0.7656 |
| **MONO** | **(10^3^/µL)** | 0 ± 0 | 0.24 ± 0.1 | 0.24 ± 0.13 | 0.9818 |
|  | **(%)** | 0 ± 0 | 4.74 ± 2.39 | 3.89 ± 1.69 | 0.5111 |
| **Lymph** | **(10^3^/µL)** | 4.75 ± 0.81 | 3.33 ± 0.91 | 3.76 ± 1.28 | 0.5696 |
|  | **(%)** | 90 ± 3.39 | 62.37 ± 4.8 | 61.47 ± 7.82 | 0.8320 |
| **CD4+ T cells (%)** | | 12.35 ± 3.46 | 8.26 ± 1.85 | 10.8 ± 1.5 | ***0.0211*** |
| **CD8+ T cells (%)** | | 9.35 ± 1.2 | 6.35 ± 0.67 | 7.89 ± 1.15 | ***0.0091*** |
| **CD19+ B cells (%)** | | 60 ± 8.49 | 24.93 ± 4.72 | 28.91 ± 9.18 | 0.3146 |
| **F4/80+ macrophages (%)** | | 4.15 ± 0.21 | 10.42 ± 2.08 | 8.36 ± 2.46 | 0.1179 |
| **Granulated cells, based on FSC/SSC (%)** | | 8.48 ± 3.5 | 41.36 ± 8.21 | 36.73 ± 8.74 | 0.3313 |
